# Assembly of a 3D Cellular Computer Using Folded E-Blocks 

**DOI:** 10.3390/mi7050078

**Published:** 2016-04-28

**Authors:** Shivendra Pandey, Nicholas J. Macias, Carmen Ciobanu, ChangKyu Yoon, Christof Teuscher, David H. Gracias

**Affiliations:** 1 Department of Chemical and Biomolecular Engineering, The Johns Hopkins University, Baltimore, MD 21218, USA; shivendra@jhu.edu; 2 Department of Engineering and Computer Science, Clark College, Vancouver, WA 98663, USA; 3 Department of Electrical and Computer Engineering, Portland State University, Portland, OR 97207, USA; ccarmen@pdx.edu; 4 Department of Materials Science and Engineering, The Johns Hopkins University, Baltimore, MD 21218, USA; yck0825@gmail.com

**Keywords:** cell matrix, architecture, self-configurability, self-assembly

## Abstract

The assembly of integrated circuits in three dimensions (3D) provides a possible solution to address the ever-increasing demands of modern day electronic devices. It has been suggested that by using the third dimension, devices with high density, defect tolerance, short interconnects and small overall form factors could be created. However, apart from pseudo 3D architecture, such as monolithic integration, die, or wafer stacking, the creation of paradigms to integrate electronic low-complexity cellular building blocks in architecture that has tile space in all three dimensions has remained elusive. Here, we present software and hardware foundations for a truly 3D cellular computational devices that could be realized in practice. The computing architecture relies on the scalable, self-configurable and defect-tolerant cell matrix. The hardware is based on a scalable and manufacturable approach for 3D assembly using folded polyhedral electronic blocks (E-blocks). We created monomers, dimers and 2 × 2 × 2 assemblies of polyhedral E-blocks and verified the computational capabilities by implementing simple logic functions. We further show that 63.2% more compact 3D circuits can be obtained with our design automation tools compared to a 2D architecture. Our results provide a proof-of-concept for a scalable and manufacture-ready process for constructing massive-scale 3D computational devices.

## 1. Introduction

A significant difference between present day electronic devices, such as von Neumann microprocessors and biological computational devices, such as the brain, is the spatial arrangement of their basic building blocks. At both the transistor, as well as the chip scale, conventional integrated circuits and electronic devices are composed of components that are arranged in inherently two dimensions (2D), although there may be several layers of processing elements and connections. In contrast, the brain consists of on the order of one hundred billion neurons that are interconnected in a truly three-dimensional (3D) architecture. As compared to a 2D architecture, a true 3D architecture leads to shorter average path lengths in the interconnect network and offers the potential for higher interconnect densities which can lead to increased circuit complexity, computational speed, and power efficiency [1,2,3,4]. A number of approaches have been developed to interconnect electronic components ranging from transistors to microchips in 3D. These include conventional approaches, such as monolithic integration and die and wafer stacking [5,6,7,8,9,10,11,12], which have advanced significantly in the industry with noted challenges associated with precise alignment of devices and throughput [13]. It should be noted that wafer stacking is a serial process and, as a consequence, the extent of three-dimensionality that could be achieved is limited; hence, wafer stacking actually achieves a 2.5D architecture, and not a truly 3D architecture. In addition, wafer stacking comes with its own challenge: the alignment of the vias [14,15]. 

An alternative approach to top-down design is the biologically-inspired approach of self-assembly. Indeed, there has been a long-standing effort in the research community to build 3D electronic devices and reconfigurable modular robots using self-assembly [16,17,18]. In self-assembly, the focus is on generating smart building blocks that can self-organize into a functional structure. Various building blocks, such as molecules or nanoparticles, have been proposed. However, these approaches have limitations, such as the relative thermal fragility and low electrical conductance of organic molecules or the inability to precisely define patterned transistors using nanoparticles. Hence, there is an urgent need for scalable and mass-producible processes that bridge the gap between existing chip manufacturing strategies and self-assembly methodologies. 

**Figure 1:**
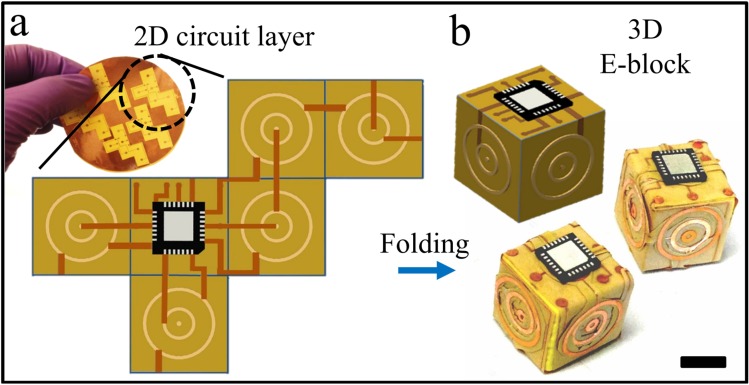
Layout for folded E-blocks. (**a**) Schematic and experimental photograph illustrating the two dimensional (2D) mapping of circuit layers designed for a three dimensional (3D) cubic E-block; (**b**) Schematic and photograph of an experimentally realized 3D folded E-block monomer. Scale bar is 5 mm.

Here, we describe a new framework for the creation of 3D devices using polyhedral folded electronic blocks (E-blocks) and demonstrate proof-of-concept for the fabrication of fully functional 3D computational devices. The approach for fabricating E-blocks by folding is illustrated in Figure 1. We describe the software and hardware foundations for implementing this concept using cellular computational chips and discuss the challenges with fabrication, interconnect routing, assembly, defect mitigation, and programming. Based on these results, and prior literature on self-folding and self-assembly of polyhedra, we argue that this approach is manufacture-ready in a high-throughput and fully self-directed manner to create truly 3D, very large scale integration of next generation computational devices. While we realize that the self-assembly of E-blocks will have technical challenges such as the generation and electrical testing of defect-free organized aggregates with appropriate wiring and pin-on-pin connectivity, work to date suggests that with the addition of the self-configurable, fault tolerant cell matrix architecture, most of the hardware related challenges can be addressed.

## 2. Results and Discussion

This work consists of two parts: (a) software foundations for the development of a self-configurable and fault-tolerant 3D tool chain and (b) hardware foundations for a fabrication method to manufacture folded E-blocks.

### 2.1. Software Foundations

Although cellular systems can be attractive for 3D computers, there are significant challenges for the software, especially in the handling of defects that arise during the hardware assembly process. Prior approaches for handling defects in cellular computational devices include increasing redundancy or implementing architecture that is inherently defect-tolerant, such as scale-free networks. Such networks can still robustly communicate data between nodes despite high failure rates [19,20]. The core of our approach consists in extending the cell matrix architecture from a 2D to a 3D architecture, as introduced earlier [21,22,23]. In order for this approach to work, a balance must be struck. The cells need to be simple enough that they can be fully tested with a small, well-defined set of test sequences; yet they must be powerful enough to be able to autonomously interact with neighbors (thus reducing reliance on a central, external control system). Cell matrix strikes this balance by using an extremely simple atomic unit, which has been endowed with the ability to read and write neighbors’ configurations.

This mechanism supports the development of powerful circuit behaviors. For example, in testing cells for defects, a classic issue is the possibility that the testing circuitry itself may be defective. In a cell matrix, the testing circuitry can be synthesized from the same cells that are being tested. Simple test sequences (generated from an external, trusted source) are used to first test a small number of manufactured cells one at a time. Once those cells are known to be good, they can be configured into a larger, parallel test circuit. As this test circuit discovers more perfect cells, it expands into a larger, more-parallel circuit, which is able to test more cells in parallel. This approach has a four-fold advantage: (1) it requires only a small number of known-to-be-good initial cells; (2) it grows geometrically, testing *O*(*n*^3^) cells in *O(n)* steps; (3) the circuit can continue to grow despite the presence of scattered defects (up to a critical defect density); and (4) it can operate with minimal need for external control/intervention [22,23]. 

**Figure 2:**
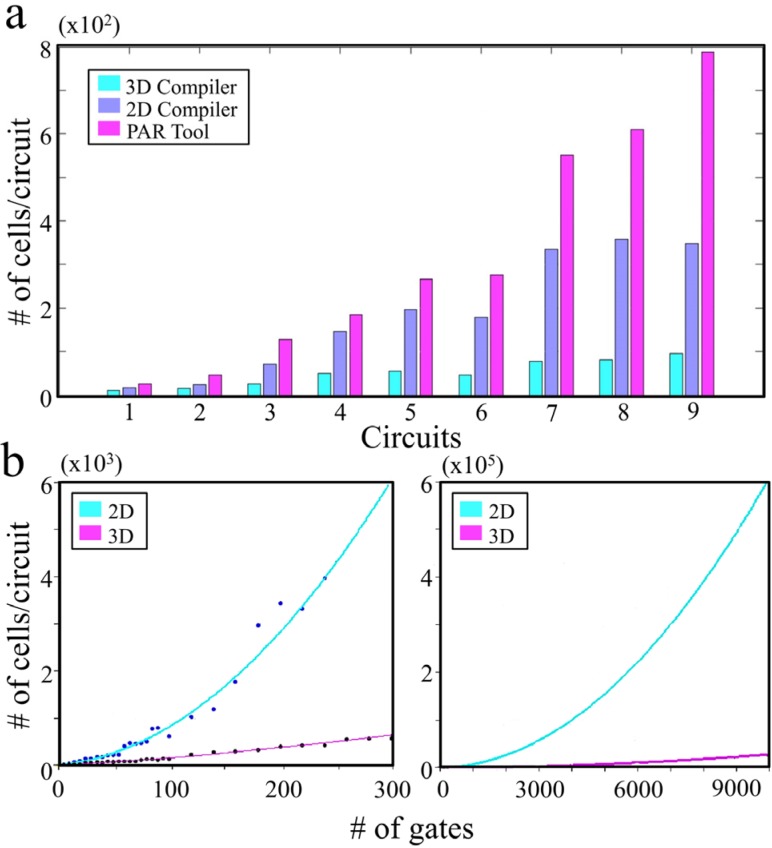
Simulation results for comparison of performance for 2D *vs.* 3D integration of gates. (**a**) 2D *vs.* 3D cell matrix compilers and PAR Tool (Perl Archive Toolkit) performance comparison; (**b**) comparison of the number of cells required for the same performance when the gates are integrated in 2D and 3D. Both 2D and 3D compilers were programmed to perform routing with A* algorithm, where shortest distance between two nodes are computed to determine the lowest cost of a function [24]. Placement of cells was computed using neural and force directed methods where a 2D cell matrix utilized square cell matrix topology, and, in 3D, the compiler was configured to work on a six-sided cell matrix topology.

The self-configurability of the matrix can also augment the physical self-assembly of E-blocks. For example, the physical orientation of assembled blocks relative to an external orientation is essentially unpredictable. However, an initial software-driven introspection process—similar to the defect-detection process described above—can be used to determine, one-by-one, the orientation of a small set of cells near the edge of the assembly. Once those blocks’ orientation is known, programming sequences can be adjusted, and the blocks can be used to build a larger orientation-detection circuit, whose testing-capability grows geometrically over time [22,23]. With simulations, we show that a 63.2% greater compactness over a 2D integration can be achieved with our 3D architecture (Figure 2). 

### 2.2. Hardware Foundations

For cellular 3D computers, there are significant challenges in implementing the hardware in practice. Hardware unit cells can be composed of biological matter, such as neurons or nucleic acids, to perform computations, but these cannot be easily used for practical computational devices for higher order computations [25,26,27,28]. The highlight of our approach is the utilization of polyhedral E-blocks as computing cells with attractive characteristics, such as manufacturability, scalability, controlled assembly, density, robustness, and the ability to include porosity to circulate fluids to reduce heating of the elements. We show proof-of-concept with larger blocks mainly due to the relatively high costs of fabricating smaller Complementary Metal–Oxide–Semiconductor (CMOS) enabled units in an academic setting. 

To our knowledge, the first self-folded E-block with patterned connector pads and single crystal silicon faces was demonstrated by Gracias *et al.* [29]. Since then, the creation of self-folded polyhedra using a high throughput and fully manufacturable process with metals, dielectrics, and semiconductors for sizes as small as 100 nm has been demonstrated [30,31,32,33]. Hence, the polyhedral E-block approach could be implemented at smaller scales and with high throughput fabrication approaches, albeit at a higher cost but readily achievable in an industry setting, and consequently represents a manufacture-ready process. Conceptually, a folding approach is also attractive because there already exists a vast infrastructure for patterning 2D CMOS devices. Folding can readily transform 2D patterned CMOS circuits into 3D curved and polyhedral devices in a high throughput manner [34]. Hence, E-blocks with surfaces patterned with a variety of electronic modules ranging from transistors to chips to interconnect and solder bumps can be readily patterned in all three dimensions in a highly parallel manner. To our knowledge, such high throughput patterning of E-blocks cannot be achieved by any other approach. 

Further, polyhedral E-blocks are attractive for self-assembly because the properties of polyhedra and their 3D tiling lattices have been extensively studied since the days of Plato over 2000 years ago [35]. Polyhedral units have been previously used for forming self-assembled functional electrical connections and periodic metamaterials [18,36]. Due to the vast knowledge of the geometric properties of these shapes, they offer unprecedented opportunities for the assembly of electronic devices. The assembly networks of polyhedral blocks can also be made both symmetrically and asymmetrically depending on the kind of polyhedral shape. For example, the tiling of polyhedra, such as cubes or truncated octahedra, results in symmetric networks while that of rectangular parallelepipeds or square pyramids results in asymmetric ones. In addition, porous networks can be synthesized using truncated octahedra, cuboctahedra, or truncated cubes, and the porosity can enable fluidic or convective cooling of 3D electronic networks, which is important for practical operation. In this study, we focus on building and configuring cube-shaped E-blocks for illustrating models of computational devices. 

### 2.3. Design of E-blocks and the Computer

**Figure 3:**
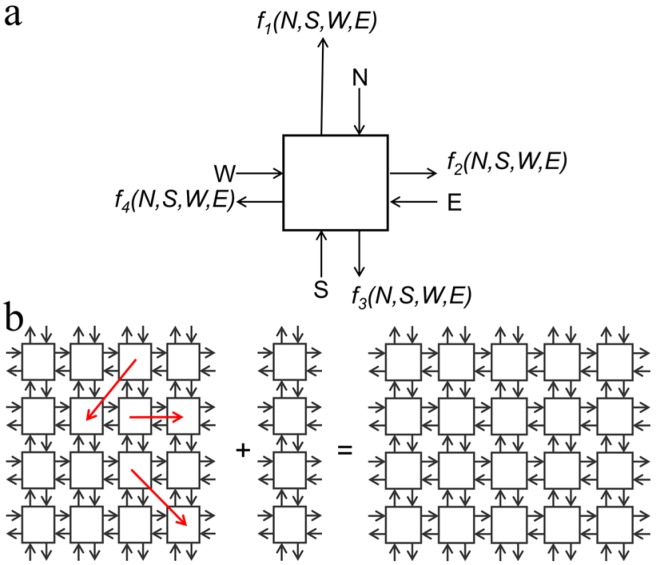
Architecture of a self-configurable and scalable 2D cell matrix. (**a**) Design of a single unit cell with input and output on each side. The cell receives information from neighbor cells, processes it and then feeds its output back to neighboring cells to configure the circuit, (**b**) a cell matrix is perfectly scalable. Adding a 4 × 1 matrix to a 4 × 4 matrix results into a 4 × 5 cell matrix. Red arrows show that each cell can interact with other cells independently and thus provide self-configurability to the matrix.

**Figure 4:**
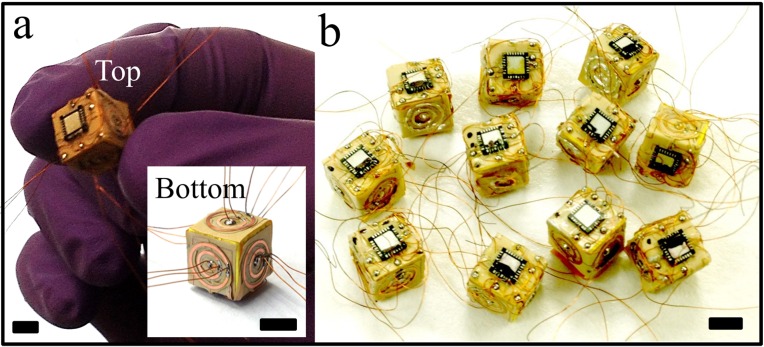
Fabrications of milli-E blocks. (**a**) Flexible 2D maps of circuits layers were cutout, folded, and mounted on the cube with a commercial microchip on top and interconnected Cu wires on each face using PCB (Printed Circuit Board) photolithography; (**b**) potentially mass-producible E-blocks were prepared with interconnecting wires to establish connections to the chip and circuit layers. All scales are 5 mm.

**Figure 5:**
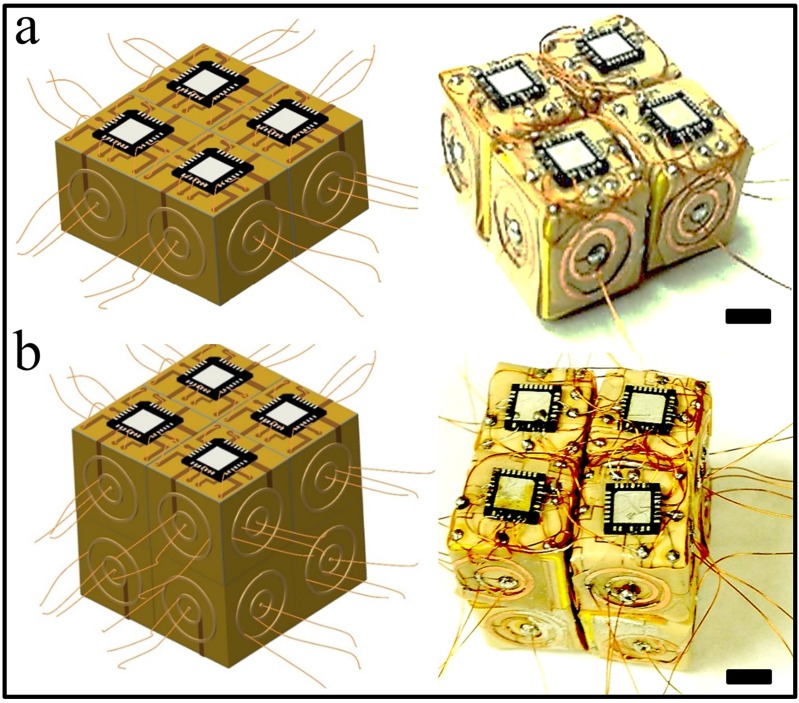
Higher order assembly of E-blocks to create 3D cellular computer. (**a**) Schematic and experimental illustration of a tetramer (2 × 2 assembly) and (**b**) an octamer (2 × 2 × 2 assembly) 3D computer. All scales are 5 mm.

The architectural design of a 3D computer presented in this paper is based on the aforementioned self-configurable inherently defect- and fault-tolerant cell matrix. Briefly, a 2D cell is a four-sided digital element that has two inputs and outputs on each side. One set of inputs look up outputs from the internal truth table of the cell, and the other set of inputs is used to read and update the truth table of that cell. After processing the data, the cell feeds the output to its neighboring cells. Thus, each cell behaves as an independent digital circuit while processing the data, which is needed to differentiate between functional and non-functional (defective) cells in the matrix. Once functional cells are identified, defective cells are excluded and functional cells interact with one another independently and generate a self-configurable fault-tolerant circuit. In addition to defect- and fault-tolerant self-configurability, cell matrix is scalable irrespective of matrix size (Figure 3). The design of a 3D computer using 3D cells is simply an extension of a 2D cell matrix. In a 2D cell matrix, a unit cell is a four-sided cell whereas in a 3D cell matrix, each unit which is a cubic E-block in this case is a six-faced 3D cell. In our experiments, these functional cubic cell units were assembled to create a 3D cell matrix of the dimer, tetramer, and octamer shapes (Figure 4 and Figure 5).

Although the eventual goal of this work is to build self-assembled 3D computers at a miniaturized scale, we made the following simplifications to illustrate proof-of-concept: (a) instead of patterning the target digital circuitry (e.g., the cell matrix architecture) onto a 2D substrate, we chose to emulate the cell matrix using a conventional microprocessor. This is an imperfect approximation, as the cell matrix’s hardware operation is parallel, whereas a software simulation on a conventional CPU is inherently sequential. Nonetheless, this allowed us to explore the configuration of a 3D cell matrix implemented via a collection of E-blocks; (b) since the only available commercial packaged microprocessor was physically large (a few mm in length and width), the E-blocks also needed to be large as well. This meant that hand-folding and assembly rather than self-folding and self-assembly was required; (c) In a 3D cell matrix comprised of six-sided cells, each cell requires seven connections (two data/two control signals, one clock signal, plus power and ground) to each neighboring cell. Since the E-blocks were manually assembled, creating seven conductors per face was not feasible. Instead, we chose to use only three conductors: power, ground, and a single shared (time-multiplexed) signal line (which carried the two data/two control signals and the clock). This is another approximation, and a departure from the inherent dataflow behavior of the cell matrix, but it presents a useful step toward the higher-level design.

One of the challenges in self-assembly is that the tiling is generated via programmed interactions of the blocks and hence patterns and the blocks themselves must encode this information. In order to facilitate high yielding self-assemblies, it is necessary to build redundancy and create funnel shaped potential interactions between blocks. While a variety of interactions such as solder based surface tension forces [18] or magnetic connectors [11,16] could be utilized for self-assembly, these principles stay the same. Here, we assembled aggregates using solder based interactions and we used two concentric circular connectors on each face to enable this interaction (see layout in Figure 1a). Our choice was based on results from prior research [37], where we discovered that geometric patterns with large overall areas, low radii of gyration, and high angular distributions result in efficient and low-defect assembly of cubes. In contrast to wafer stacking, where via alignment is required over large areas, potentially the size of a 12 or 16 inch wafer, in E-block assembly self-alignment of only two concentric circles are needed, thus greatly minimizing the challenge of providing high-fidelity alignments over large areas. Our wiring layout was designed so that a single chip on each block would connect with another chip on another block during concentric ring-on-ring assembly. 

### 2.4. Testing the Assembled E-Blocks

**Figure 6:**
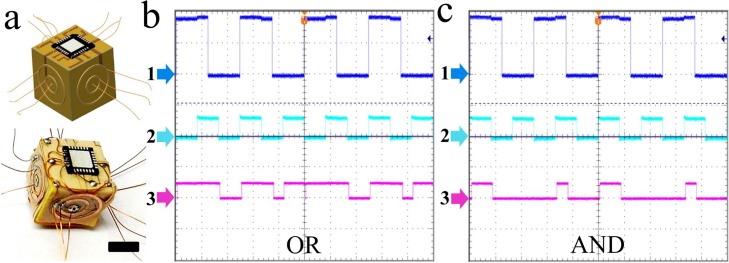
Verification of functionality of a monomer E-block. (**a**) Schematic and optical images of a wired E-block monomer. The scale bar indicates 5 mm; (**b**,**c**) experimentally recorded oscilloscope readings show the output of the monomer configured as (**b**) OR gate and (**c**) AND gate. 1 and 2 represent inputs and 3 is the output.

**Figure 7:**
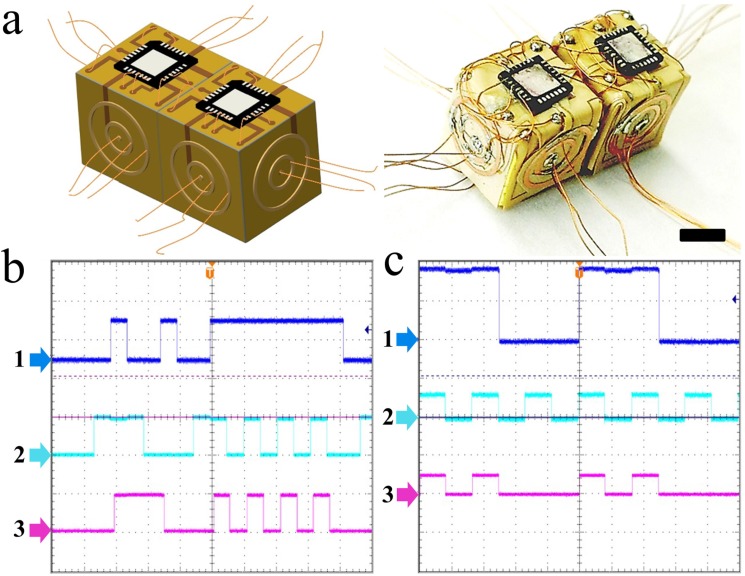
Verification of functionality of dimer E-blocks. (**a**) Schematic and optical images of dimer E-blocks. The scale bar indicates 5 mm; (**b**,**c**) experimentally recorded oscilloscope readings show the output of the dimer configured as D-latches, where 1 and 2 are LOAD and D inputs respectively, and 3 is the output. LOAD input is the enable line that provides ability to store the previous input value or load the new input value. When LOAD = 1, D-input is passed to output, when LOAD = 0, the D-input remains latched inside. Here, Both (**b**) and (**c**) are same circuit configuration but two different tests as D-latch.

To test the E-blocks, we used a testbed driver, which performs three functions: (a) it allows a host Linux machine to send 1's and 0's to the C and D inputs of the cells being tested and for outgoing bits (generated by the cells) to be displayed on a series of Light Emitting Diodes (LEDs); (b) it also allows the driving program to be disconnected, so that external hardware can supply the 1’s and 0’s to the cells being tested: and (c) it handles the time-multiplexing of D and C inputs and outputs (and clock signals) through the single-conductor signal contact on each face of the E-blocks. Time-multiplexing was employed to reduce the number of inter-block connections. In a self-assembled system built from lithographically-patterned blocks, we anticipate multiple signal connections per face, thus avoiding the need to time-multiplex signals. The testbed set-up and configuration of the E-blocks as gates and latches are shown in Figure S1. We note that we were successfully able to configure monomers and dimer as simple OR, XOR, AND gates, 2-bit adder, and D-latches for various inputs and confirmed that the folded E-blocks were fully functional (Figure 6 and Figure 7). Dimer testing showed that the circuits can be built to incorporate feedback mechanisms of functional E-blocks. 

### 2.5. Scalability and Manufacturability

As a proof-of-concept for a 3D computer, we manufactured, assembled, and tested the millimeter-scale cube-shaped E-blocks and successfully demonstrated their functionality as an AND gate, OR gate and simple 2-bit adder. By using 3D self-assembly methodologies to create nano- and microscale complex-shape 3D structures from 2D precursors and self-assembly approaches [28,29,30,31], the E-blocks could potentially be manufactured at the nano- and microscale in a high yield parallel fabrication process. In order to mitigate defects during self-assembly, optimized patterns can be introduced on the faces of E-blocks as described above [36].

## 3. Materials and Methods

### 3.1. Fabrication of E-blocks

We first mapped each layer of the desired 3D circuit diagram to a 2D layout with three layer–voltage, ground, and signal (Figure 1a). Photomasks were designed using AutoCAD and printed on transparency films. For each layer of circuitry, we spincoated a photoresist (S1827, Rohm and Haas, Philadelphia, PA, USA) at 3000 rpm onto a sheet of flexible copper-clad laminate (Pyralux^®^ FR 9110R, Dupont, NJ, USA) and baked at 115 °C for 1 min. We exposed photoresist coated sheets to UV light (365 nm) at ~180 mJ/cm^2^ through respective negative masks, developed in a developer (Microposit 351 developer, Rohm and Haas, Philadelphia, PA, USA) for 30 s, etched off the exposed copper in a saturated aqueous solution of ferric chloride, rinsed the patterned sheets with deionized water (DI H_2_O) and dried with N_2_ gas. Unexposed photoresist was removed by acetone. After patterning these circuit layers, we cut them out, aligned one on the top of the other, and attached a 4 mm × 4 mm PIC16F1827 (PIC: Peripheral Interface Controller) microchip and then folded them by hand into a shape of cube. For our proof-of-concept, we used the PIC microchip simply to emulate the functionality of a cell matrix. We mounted these folded layers onto the solid cubes and completed the connection to make functional E-block units. The connector rings on the E-blocks were coated with solder and then assembled to create the desired cellular assembly where each concentric ring overlapped that on a neighbor. 

### 3.2. E-blocks as Gates, D-latches and a 2-Bit Adder

In the test setup shown in Figure S1, there are two large PIC chips at the top of the board: these are simulating the chips in the dimer. The switches in the lower right can supply inputs to the cells, and the outputs are shown on the LEDs in the upper-right. The board is driven via a USB connection (top left) and commands are processed by the Terminal IO (TIO) unit, which is the silver chip in the lower right. The two yellow chips are the Interface (INT) units, which handle the time-multiplexing/demultiplexing for communicating with the cells. The monomer was soldered to a set of wires to allow connection to the testbed driver and the dimer was attached to connecting wires via magnetic connectors. A complete lab set-up used to test these E-blocks is shown in Figure S2. 

For each test, we used a command-line driver to program the E-blocks. For the monomer, this requires setting a block’s C input to 1; then, ticking the system clock 768 times while setting the D input to 1 or 0 prior to each tick; and then returning the C input to 0. For the dimer, the above process is performed three times: first, to allow block 0 to configure block 1; then, to actually configure block 1 to its desired configuration; and then to reconfigure block 0 to its desired configuration. The complexities of this are handled by the command-line driver, thus allowing simple specification of the blocks’ desired configuration. Note, however, that in the case of the dimer, all configurations were performed using a single signal (SIG) contact on one face of a single block. In more complex arrangements, two SIG contacts must be used. In the case of larger circuits, three SIG contacts (on three adjacent blocks) must be used. Note that access to three SIG contacts suffices to enable programming of arbitrarily-large collections of blocks.

For a simple test, a single block was configured as a two-input AND and XOR gate. The inputs were driven by a function generator, and the output was observed on an oscilloscope. The two inputs are on top (yellow and blue), and the output is on the lower trace (purple). The E-block performed perfectly, implementing the AND and XOR functions (Figure S3). An E-block was also configured as a 2-bit adder. The circuit is designed to add two 2-bit numbers (A1 A0 and B1 B0) and produce a 3-bit sum (S2 S1 S0) where A0, B0 and S0 are the least significant bits (LSBs). The traces are ordered as indicated on the picture. The E-block performed perfectly as a 2-bit adder (Figures S4 and S5).

The dimer was used in some single-block tests, but the more-interesting use of the dimer is in circuits that employ both blocks. The main benefit of two blocks *vs.* one is that circuits can be built that incorporate feedback, *i.e.*, circuits that exhibit memory. For this test, the dimer was configured as a 2-cell D latch, having two inputs (LOAD and D) and one output (Q). The dimer was configured using the three-step programming sequence described above: all configurations were performed via a single face on one of the blocks. In all cases, the circuit worked perfectly. In this first test, the LOAD and D inputs were driven by the command-line driver. As shown in Figure S6, the lower trace is the LOAD line; the middle trace is D; and the upper trace (orange) is the output Q. As can be seen, when LOAD = 1 the D input is passed to the output; and the D value present when LOAD drops is latched inside the device. The circuit output was also monitored using an oscilloscope. In these tests, the LOAD line is on top (yellow); D is the middle trace (blue); and the output Q is on the bottom (purple). Inputs were again driven by the command-line driver. In all these tests, the monomer and the dimer performed perfectly, implementing the intended functions exactly as they were configured to do.

## 4. Conclusions

The eventual goal of this work is to: (a) lithographically pattern micro-nanoscale 2D digital circuit patterns; (b) have these patterns self-fold (in parallel) into 3D polyhedra (E-blocks); and (c) have those polyhedra self-assemble into 3D collections of E-blocks which function as computational devices. Unfortunately, step (a) requires large up-front costs and non-recurring engineering charges, which were outside the budget of this effort. Instead, we opted for a proof-of-concept of the process, by utilizing a commercial off-the-shelf packaged 4 mm sized microprocessor which required large E-blocks and the process was implemented manually, but we note that all elements of the process such as self-folding of smaller units and pin-on-pin aggregative solder assembly have been independently verified, and hence this represents a manufacture-ready process. 

Despite the presence of a complete microprocessor in each E-block, it was pre-programmed prior to mounting, and not re-programmed after assembly. Consequently, the microprocessor was only used to emulate the fixed cell matrix architecture. However, the same architecture allows the implementation and re-implementation of digital circuits using the matrix's self-configurability. This means that when E-blocks are eventually produced from micropatterned silicon that directly implements the cell matrix architecture, the system's behavior will still be modifiable via the ability to configure/re-configure/self-configure cells within the system. This is particularly important since when E-blocks self-assemble, the relative orientation of the blocks is effectively unknown. By arranging inter-block connections in a series of concentric circles, face-to-face communication is ensured regardless of orientation. While cells rely on an understanding of their orientation within the matrix (since faces are specified in terms of +/−*X*, +/−*Y*, +/−*Z*), a collection of cells can work cooperatively to determine the orientation of cells within the collection [23].

Given an assembled collection of 3D E-blocks, it can be configured to implement any desired digital circuit, including combinational logic, state machines, a CPU, a cluster of CPUs, *etc.* This configuration can be done using very conventional design techniques (external to the 3D assembly), but, for very large collections, this may not be feasible. Instead, self-organizing principles can be employed, such as those described in [38]. By designing a blueprint or a target circuit, the 3D assembly can be tiled with identically-configured sub-blocks ("supercells"), each containing a copy of the blueprint, plus the necessary machinery for a variety of functions, including (a) testing of regions of E-blocks for errors; (b) parallel configuration of supercells from sets of defect-free E-blocks; (c) interpreting the blueprint; (d) self-locating (determining (*X*,*Y*,*Z*) location) within the collection of supercells; (e) mapping of communication paths from supercell to supercell; and (f) differentiation of supercells into desired functions in order to implement the target circuit.

In conclusion, the self-assembly of a 3D self-configuring cell matrix is significant in several ways—(a) the parallelism of self-folding of E-blocks means large numbers of blocks can be readily manufactured; (b) the fault-tolerance of the architecture means that even with a low yield, useful circuitry can still be built via use of introspective assembly techniques [23]; (c) digital circuitry that fully exploits all three dimensions that remain elusive under conventional manufacturing, whereas 3D E-blocks provide a manufacture-ready path to large-scale 3D assemblies; (d) target circuits can be specified/modified post-manufacture, allowing the physical substrate to be manufactured in a fixed, pre-defined way regardless of the specific intended use; and (e) customization of the system's behavior is specified via reconfiguration of hardware (which is inherently parallel), *vs.* reprogramming of software (which is primarily sequential), thus allowing potentially higher performance.

Our work is thus broadly relevant for future and emerging 3D computers that may be self-assembled from a massive number of imperfect building blocks.

## Supplementary Materials

The following are available online at http://www.mdpi.com/2072-666X/7/5/78/s1, Images and testing data for the E-blocks and their aggregates Figure S1: Testbed driver used to test the E-blocks. Figure S2: Test driver set up used to test E-blocks. (a) Complete testbed set up showing oscilloscopes. The oscilloscope in the upper middle is a 4-channel Tektronix scope; the function generator is in the upper right. The testbed driver is in the lower middle, and the E-blocks are behind the testbed; (b) a zoomed in optical image of the oscilloscope showing test results. Figure S3: Oscilloscope showing experimental results of the E-blocks. E-blocks configured as (a) an AND gate, (b) an XOR gate. Top two signals (yellow and blue) represent the inputs and the bottom signal (purple) represents the output. Figure S4: Testing the E-blocks using the test bed. Four switches at the bottom left represent binary inputs 10, 10 and the three encircled LEDs on the right left represent output as 100, thus configured as a 2-bit adder. Figure S5: Photograph of the oscilloscope screen showing experimental result of the E-block tests a 2-bit adder. Two 2-bit numbers (A1 A0 and B1 B0) and produce a 3-bit sum (S2 S1 S0) where A0, B0 and S0 are the least significant bits (LSBs). The traces are ordered as indicated on the picture. The block performed perfectly as a 2-bit adder. Figure S6: Experimental result of dimer configured as a D-latch. The dimer was configured such that the circuits exhibit memory. For this test, the dimer was configured as a 2-cell D latch, having two inputs (LOAD and D) and one output (Q). When LOAD = 1 the D input is passed to the output Q, and the D value present, when LOAD drops, is latched inside the device.
