# Supplementary material for: Assembly of a 3D Cellular Computer Using Folded E-Blocks
Source: Micromachines (Basel). 2016 Apr 28;7(5):78. doi: 10.3390/mi7050078 (PMC6189794; doi:10.3390/mi7050078)
Supplement: Supplementary file 1 [file micromachines-07-00078-s001.pdf]

Supplementary Materials: Assembly of a 3D Cellular Computer Using Folded E-Blocks

Shivendra Pandey [1], Nicholas J. Macias [2,*], Carmen Ciobanu [3], ChangKyu Yoon [4], Christof Teuscher [3,*], David H. Gracias [1,4,*]

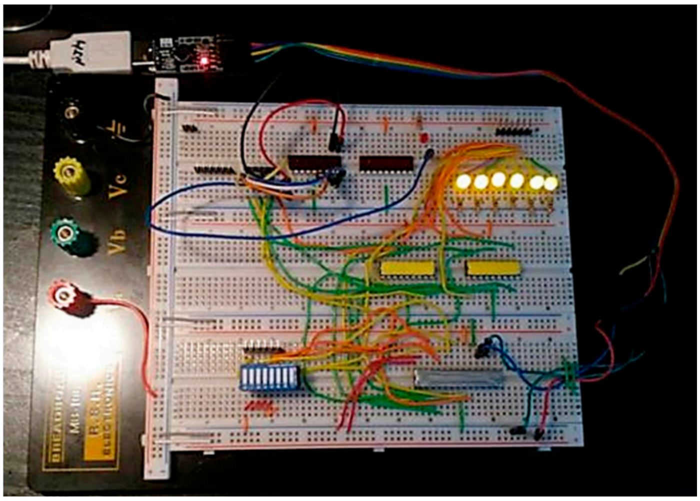

Figure S1. Testbed driver used to test the E-blocks.

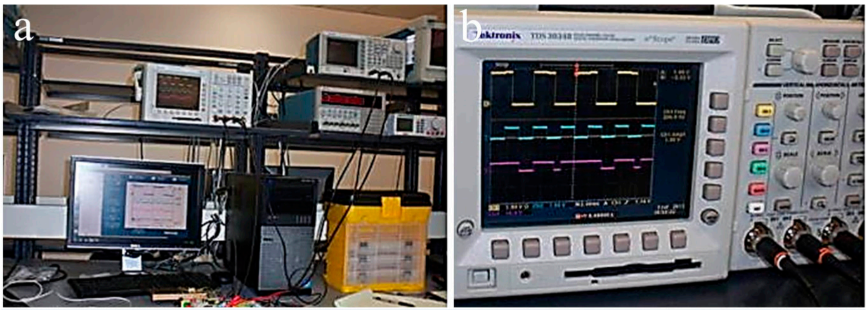

Figure S2. Test driver set up used to test E-blocks. (**a**) Complete testbed set up showing oscilloscopes. The oscilloscope in the upper middle is a 4-channel Tektronix scope; the function generator is in the upper right. The testbed driver is in the lower middle, and the E-blocks are behind the testbed; (**b**) a zoomed in optical image of the oscilloscope showing test results.

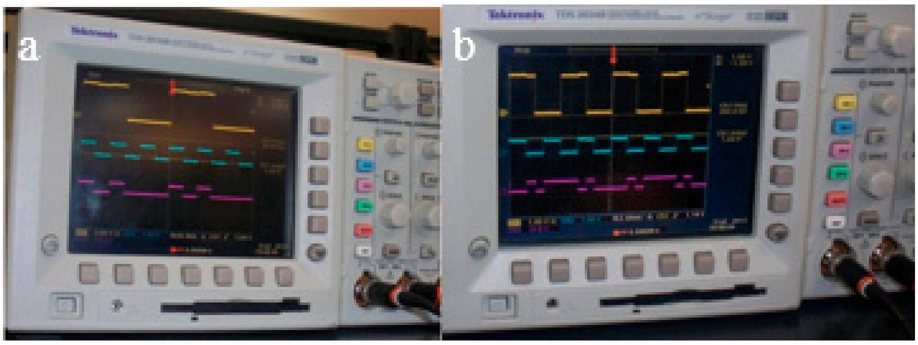

Figure S3. Oscilloscope showing experimental results of the E-blocks. E-blocks configured as (**a**) an AND gate, (**b**) an XOR gate. Top two signals (yellow and blue) represent the inputs and the bottom signal (purple) represents the output.

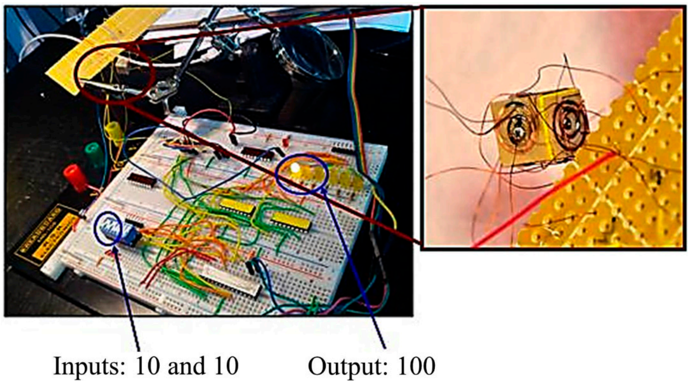

Figure S4. Testing the E-blocks using the test bed. Four switches at the bottom left represent binary inputs 10, 10 and the three encircled LEDs on the right left represent output as 100, thus configured as a 2-bit adder.

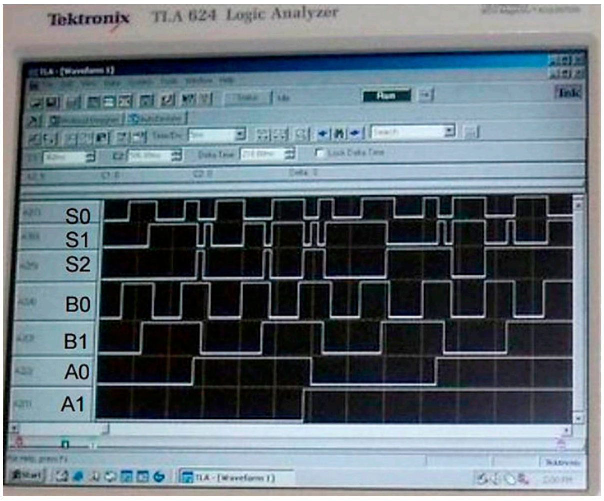

Figure S5. Photograph of the oscilloscope screen showing experimental result of the E-block tests a 2-bit adder. Two 2-bit numbers (A1 A0 and B1 B0) and produce a 3-bit sum (S2 S1 S0) where A0, B0 and S0 are the least significant bits (LSBs). The traces are ordered as indicated on the picture. The block performed perfectly as a 2-bit adder.

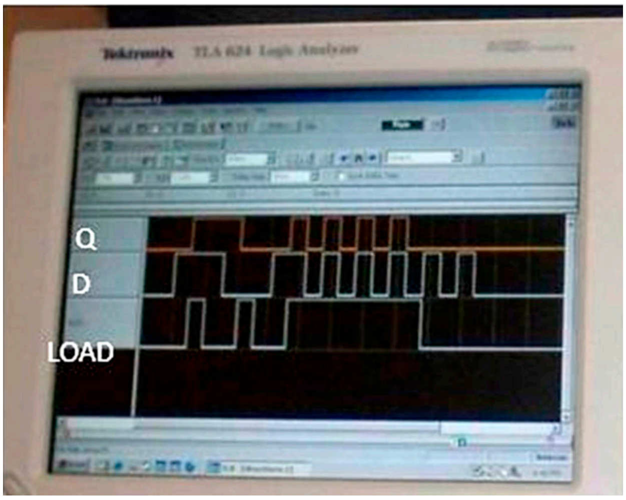

Figure S6. Experimental result of dimer configured as a D-latch. The dimer was configured such that the circuits exhibit memory. For this test, the dimer was configured as a 2-cell D latch, having two inputs (LOAD and D) and one output (Q). When LOAD = 1 the D input is passed to the output Q, and the D value present when LOAD drops is latched inside the device.
